# Benzyldimethyldodecyl Ammonium Chloride-Doped Denture-Based Resin: Impact on Strength, Surface Properties, Antifungal Activities, and In Silico Molecular Docking Analysis

**DOI:** 10.3390/jfb15100310

**Published:** 2024-10-18

**Authors:** Sarah Aldulaijan, Raghad Alruwili, Rawan Almulaify, Fatimah A. Alhassan, Yousif A. Al-Dulaijan, Faris A. Alshahrani, Lamia Mokeem, Mohammed M. Gad, Mary Anne S. Melo, Abdulrahman A. Balhaddad

**Affiliations:** 1 Chemistry Department, College of Science, Imam Abdulrahman Bin Faisal University, P.O. Box 1982, Dammam 31441, Saudi Arabia; 2 College of Dentistry, Imam Abdulrahman Bin Faisal University, P.O. Box 1982, Dammam 31441, Saudi Arabia2180006032@iau.edu.sa (F.A.A.); 3 Department of Substitutive Dental Sciences, College of Dentistry, Imam Abdulrahman Bin Faisal University, P.O. Box 1982, Dammam 31441, Saudi Arabiammjad@iau.edu.sa (M.M.G.); 4 Department of Restorative Dentistry, College of Medicine and Dentistry, Riyadh Elm University, Riyadh 13244, Saudi Arabia; 5 Department of Comprehensive Dentistry, School of Dentistry, University of Maryland, Baltimore, MD 21201, USA; 6 Department of Restorative Dental Sciences, College of Dentistry, Imam Abdulrahman Bin Faisal University, P.O. Box 1982, Dammam 31441, Saudi Arabia

**Keywords:** acrylic, antifungal, bioactive, denture, resin

## Abstract

*Candida albicans* (*C. albicans*) adhering to denture-based resins (DBRs) is a known cause of denture stomatitis. A new approach to prevent denture stomatitis is to include antimicrobial substances within DBRs. Here, we examined the mechanical performance and antifungal properties of DBRs containing benzyldimethyldodecyl ammonium chloride (C_12_BDMA-Cl) as an antimicrobial compound. C_12_BDMA-Cl is a quaternary ammonium compound, and its antifungal properties have never been investigated when combined with dental acrylic resin. Therefore, we modified a commercially available heat-polymerized acrylic DBR to contain 3 and 5 wt.% of C_12_BDMA-Cl. Unmodified DBR was used as a control group. Specimens were prepared using the conventional heat processing method. The specimen’s flexural strength, elastic modulus, microhardness, and surface roughness were evaluated. *C. albicans* biofilm was grown on the specimens and assessed via colony-forming units (CFUs) and scanning electron microscopy (SEM). In silico molecular docking was applied to predict the potential C_12_BDMA-Cl inhibition activity as an antifungal drug. The 3% C_12_BDMA-Cl DBR demonstrated antifungal activities without a deterioration effect on the mechanical performance. SEM images indicated fewer colonies in DBR containing C_12_BDMA-Cl, which can be a potential approach to managing denture stomatitis. In conclusion, C_12_BDMA-Cl is a promising antifungal agent for preventing and treating denture stomatitis.

## 1. Introduction

Optimal tissue coverage by the denture base is needed in removable prostheses for retention, support, and stability [1]. However, the extensive coverage of the prosthesis can make oral hygiene practices more challenging, leading to an increased likelihood of plaque and debris accumulation on the prosthesis and in the oral cavity [2]. This, in turn, can diminish exposure between the soft tissues and saliva [3]. As a result, the denture’s intaglio surface created an ideal environment for the adhesion of microorganisms and biofilm growth, primarily by an overgrowth of *Candida albicans* (*C. albicans*) [4]. Following certain inducing factors such as poor oral hygiene, decreased salivary flow/xerostomia, and poorly fitting dentures, the microbial imbalance beneath the denture base resins (DBRs) causes the development of mucosal inflammatory lesions and denture stomatitis [5,6].

Denture stomatitis is among the most common oral inflammatory diseases, affecting between 15% and 70% of denture wearers worldwide [7]. Clinically, it appears as a diffuse or localized erythematous patch with well-defined contours where the dental prosthesis and oral cavity mucosa come into contact [8]. Patients usually present with pain, itching, burning sensations, inflammation, or the swelling of mucosal tissues covered by the denture [9]. Moreover, it can be associated with systemic diseases, such as aspiration pneumonia, pulmonary candidiasis, nutritional deficiencies, and hematological conditions [8,9,10].

To treat denture stomatitis, patients are advised to stop wearing the prosthesis to eliminate its faults, clean the existing denture, and/or fabricate a new one [11]. Unfortunately, *Candida* biofilm is polymicrobial and challenging to manage as it penetrates the DBRs through microporosities and creates a strong bond with the biomaterials [12]. Therefore, the biofilm cannot be sufficiently removed from the DBR surfaces using conventional cleaning techniques, such as mechanical brushing [12]. Chemical cleaning techniques involve submerging dentures in various cleaning agents and accessing denture base undercuts that are difficult to clean mechanically [13]. However, this may leach out certain chemicals from materials over time, deteriorating the denture’s mechanical properties [14]. Neglecting denture stomatitis may lead to systematic infection, especially among medically compromised patients [15].

Due to the high occurrence of denture stomatitis, researchers have been devoted to conducting new therapeutic strategies, such as adding or integrating various antifungal agents into the denture base to suppress the growth of microbes around the denture and prevent the disease without affecting the mechanical and physical features of the DBRs [8,12]. DBRs with bioactive properties influence the effectiveness and durability of dentures in clinical use. However, there is insufficient research on the incorporation of bioactive agents into DBRs. One bioactive agent is benzyldimethyldodecyl ammonium chloride (C_12_BDMA-Cl), a disinfectant with excellent bactericidal action on vegetative forms of bacteria and exhibiting high safety, low irritation, and low cost [16]. The effects of its addition to DBRs has never been explored. Therefore, investigating the incorporation of C_12_BDMA-Cl into DBRs as an approach to prevent the onset of denture stomatitis among denture wearers is an area that is worth investigating. We aimed here to assess the incorporation of different concentrations of C_12_BDMA-Cl into DBRs by investigating the mechanical performance, surface characteristics, and antifungal properties of C_12_BDMA-Cl-doped DBRs. We hypothesized that incorporating C_12_BDMA-Cl into DBRs would prevent *C. albicans* biofilm development without compromising the mechanical and physical properties of the material.

## 2. Materials and Methods

### 2.1. Study Design

**Figure 1:**
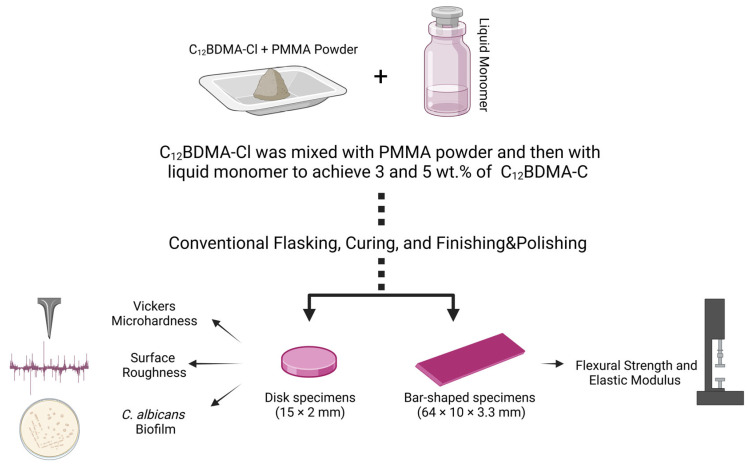
Flowchart of the study design and specimen fabrication.

This in vitro experiment evaluated the mechanical properties, surface features, and antifungal properties of a heat-polymerized acrylic DBR (Major Base 20; Major Prodotti Dentari SPA, Moncalieri, Italy) containing 3 wt.% and 5 wt.% of C_12_BDMA-Cl (Sigma-Aldrich, Saint Louis, MO, USA). A group without C_12_BDMA-Cl was set as a control group. The tested properties were assessed via flexural strength, elastic modulus, surface roughness, and microhardness. At the same time, the antifungal properties were evaluated by quantifying the colony-forming units (CFUs) of *C. albicans* over the synthesized materials. A flowchart of the study design and sample preparation is presented in Figure 1.

### 2.2. Specimens Preparation

Disk specimens (15 × 2 mm) were prepared for antifungal activities and related properties (hardness and surface roughness), while bar-shaped specimens (64 × 10 × 3.3 mm) were fabricated for the flexural strength and elastic modulus according to ISO standards (ISO recommendations (ISO 20795-1:2013) [17]. At 400 rpm for 30 min, the concentrations of C_12_BDMA-Cl after measuring were mixed with the poly methyl methacrylate (PMMA) powder. This mixing process ensured an even dispersal of the filler within the PMMA [18,19].

Sample fabrication began by creating wax specimens with the required dimensions and investing them within a metal flask. This was followed by a wax burn-out process, which created the necessary mold spaces. While the stone mold was still warm, a universal resin separator medium (Isol Major; Major Prodotti Dentari Spa, Moncalieri, Italy) was placed to the stone exteriors prior to packing the acrylic resin. The acrylic resin powder had been pre-weighed and then combined with the monomer liquid, as per the manufacturer’s instructions.

Once the resin mixture reached the dough-like stage, it was carefully packed into the mold space within the flask. The flask was then sealed and subjected to 30 min of pneumatic pressing. Finally, the acrylic resin samples were cured using a thermal curing device (KaVo Elektrotechnisches Werk GmbH, Leutkirch, Germany). The curing process involved two stages: first, the samples were heated to 73 °C for 90 min, and then the temperature was elevated to 100 °C for an additional 30 min [18]. Once the polymerization process was completed, the flasks were cooled to room temperature before being de-flasked, and the specimens were retrieved.

After the acrylic resin samples were cured, any excess material was carefully removed using a tungsten carbide bur (HM 79GX-040 HP; Meisinger, Centennial, CO, Canada). This bur was rotated at a speed of 18,000 rpm to precisely trim the specimens to the desired dimensions. Next, a digital caliper with a 0.01 mm accuracy (Neiko 01407A Electronic Digital Caliper; Neiko Tools US, LaPorte, IN, USA) was utilized to confirm that the specimen dimensions matched the specified requirements. The surface of each specimen was then polished as previously described [18,19].

### 2.3. Evaluating the Mechanical Properties of C_12_BDMA-Cl-Containing DBRs

#### 2.3.1. Flexural Strength and Elastic Modulus

After accuracy measurements, a three-point bending test in accordance with ISO standards [17] was used to assess the flexural strength (MPa) and elastic modulus (GPa). The specimens were compressed until they fractured at a 5.0 mm/min crosshead speed using a Universal Testing Machine (ElectroPlusTM E3000; Instron, Buckinghamshire, UK).

#### 2.3.2. Surface Roughness (Ra, µm)

The average surface roughness (Ra) was determined by a noncontact optical profilometer (Contour GT; Bruker Nano gmbH, Schwarzschildstrasse, Berlin, Germany). At three different locations on each specimen, an area measuring approximately (0.43 Å~0.58 mm^2^) was scanned using a regular camera at a distance of 20 Å, and the average values were computed in μm [20].

#### 2.3.3. Microhardness (VHN)

Surface hardness was tested using a Vickers tester (Wilson Hardness, ITW Test & Measurement GmbH, Shanghai, China). The hardness of each specimen was measured by applying a diamond indenter with a 25 g load for 15 s. The process was repeated three times, and the average was recorded [21].

### 2.4. The Antifungal Properties of the C_12_BDMA-Cl-Containing DBRs

The specimens were sterilized in 70% alcohol for the *C. albicans* assay, and then they were ultrasonically cleaned for 5 min in sterilized distilled water. *C. albicans* (ATCC 10231) stock was stored at −80 degrees in a refrigerator until the time of use. The sterilized specimens were immersed in Sabouraud’s dextrose broth (Acumedica Co., Manufacturers, Inc., Bellevue, WA, USA) containing 2,000,000 overnight-grown *C. albicans* cells for 48 h to evaluate mature biofilm development at 37 °C. The acrylic specimens were washed three times in phosphate-buffered saline and deposited in sterile tubes with 1 mL of normal saline, then vortexed for 10 min and then plated in Sabouraud dextrose agar. Following 48 h of incubation, the colonies were counted (CFU/mL) following previous reports [18,22].

### 2.5. Scanning Electron Microscopy (SEM) for the Biofilm

To analyze the surface structure of both unmodified and modified specimens, the specimens were fixed and sputter-coated (Quorum, Q150R ES, Edinburgh, UK) as previously described. [20,23] Then, surfaces were examined using scanning electron microscopy (SEM) (Inspect S50; FEI Company, Brno, Czech Republic) at 20 kV and a working distance of approximately 10 mm. The magnifications were taken within a range of 100 to 200× and 700 to 750×.

### 2.6. In Silico Molecular Docking Analysis

The 3D structure of C_12_BDMA-Cl was downloaded from the PubChem database [14] and checked by PyMOL software version 4.2.0 (Schrodinger Inc., New York, NY, USA) [15]. The structures of the receptors’ proteins were taken from the protein data bank (PDB) database (www.rcsb.org/pdb, accessed on 23 June 2023) [24]. The PDB IDs for the receptors are 1AI9 for Dihydrofolate reductase (DHFR) and 2QZW for candidapepsin-1 (SAPs) [25,26]. To prepare the compound and receptors’ structures for the molecular docking calculations, we used Autodock Tools (ADT) version 1.5.6 (Scripps Research, San Diego, CA, USA) [27], and the Autodock Vina server (version 16.1.0.15350) was used to run the calculations [28]. Discovery Studio Visualizer software (v16.1.0.15350. (2015)). Waltham, MA, USA) was used to investigate the binding results and generate the figures.

In addition, we used bioactive compounds as reference compounds to investigate the ability of the C_12_BDMA-Cl compound to recognize the active sites of the enzymes under study. Based on the literature, the well-known bioactive compounds that can be used as reference compounds to study antifungal drugs for *C. albicans* DHFR and SAPs enzymes are Clotrimazole and Fluconazole, respectively [29,30]. The structures of these compounds were taken from the PubChem database [31], and the docking runs were performed using the same procedure that we used to run the docking for C_12_BDMA-Cl.

### 2.7. Statistical Analysis

Data normality and distribution were checked using the Shapiro–Wilk test. One-way ANOVA and Tukey tests were utilized to compare the effects of C_12_BDMA-Cl on the mechanical properties and the reduction in *C. albicans* growth. All data were presented as the means ± standard deviations, and tests were conducted at a significance level of 0.05 using Sigma Plot 12.0; SYSTAT (Systat Software Inc. (SSI), San Jose, CA, USA).

## 3. Results

**Figure 2:**
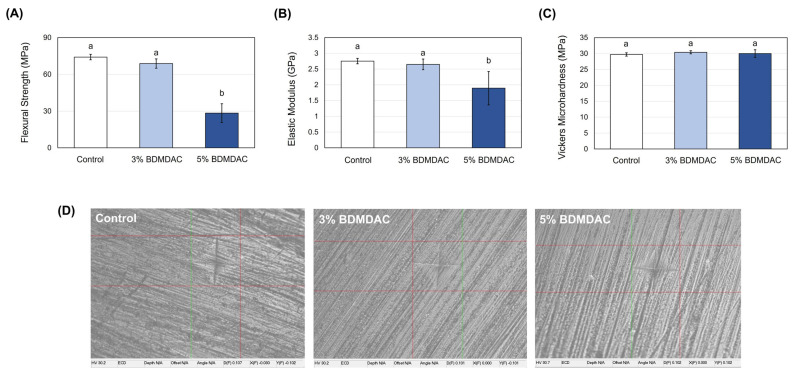
The mechanical properties assessment of the C_12_BDMA-Cl DBR groups (n = 6, mean ± SD). (**A**) Flexural strength, (**B**) elastic modulus, and (**C**) microhardness. Dissimilar letters indicate a significant difference (*p* ≤ 0.05). (**D**) Representative optical images for the microhardness test. No significant differences were found among the groups concerning their microhardness.

The 3 wt.% C_12_BDMA-Cl DBR displayed excellent flexural strength (68.78 ± 3.80 MPa) compared to the control group (74.51 ± 2.21 MPa), while the 5 wt.% C_12_BDMA-Cl DBR significantly (*p* < 0.05) decreased the flexural strength values (28.39 ± 7.63 MPa) as shown in Figure 2A. On the other hand, the elastic modulus showed no significant difference between the control group and the 3 wt.% C_12_BDMA-Cl DBR, while a slight significance (*p* < 0.05) was noticed by comparing the control group and the 5 wt.% C_12_BDMA-Cl DBR, as represented in Figure 2B. Incorporating 3 and 5 wt.% of C_12_BDMA-Cl into the DBRs did not affect their microhardness compared to the control DBR, as shown in Figure 2C.

**Figure 3:**
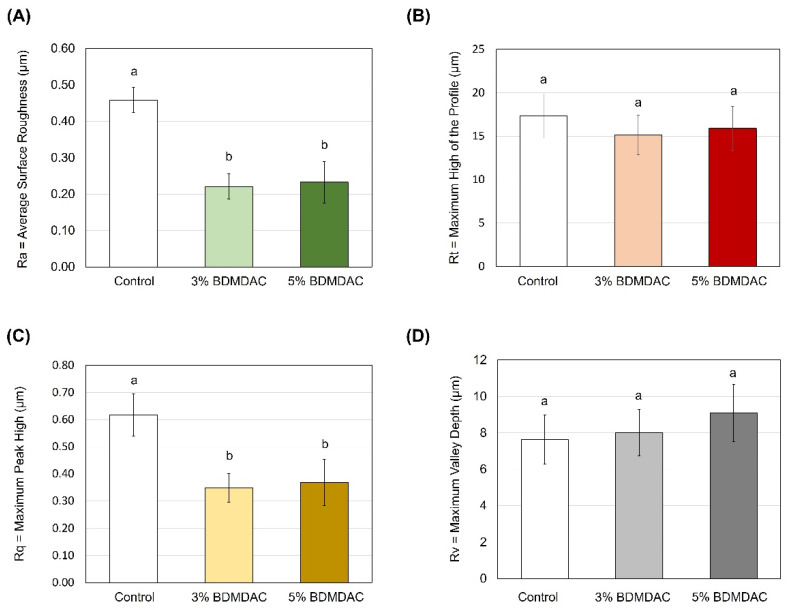
Surface roughness values of the C_12_BDMA-Cl DBR groups. (**A**) Average surface roughness, (**B**) maximum height of the profile, (**C**) maximum peak height, and (**D**) maximum valley depth (n = 10, mean ± SD). Dissimilar letters indicate a significant difference (*p* ≤ 0.05).

Figure 3 reveals that incorporating 3 and 5 wt.% of C_12_BDMA-Cl reduced the DBR’s average surface roughness (Ra) and maximum peak high (Rq) compared to the control (*p* ≤ 0.001). No significant differences were observed when the maximum height of the profile (Rt) and maximum valley depth (Rv) were compared among the groups.

**Figure 4:**
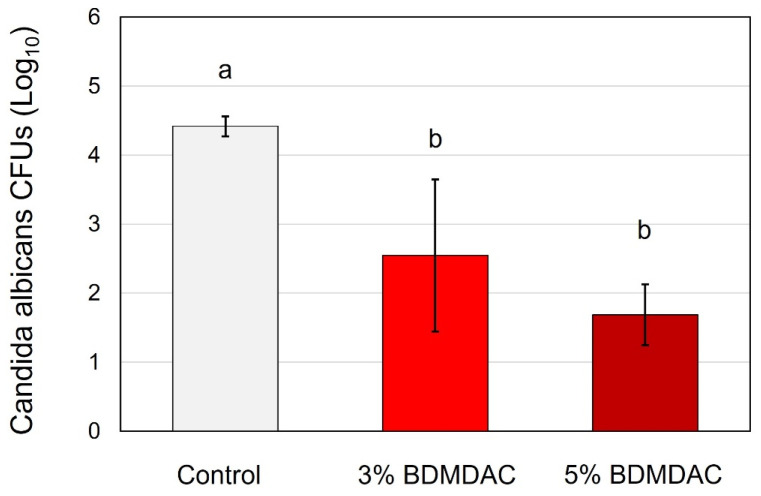
The antifungal properties of the C_12_BDMA-Cl DBR groups (n = 9, mean ± SD). Dissimilar letters indicate a significant difference (*p* ≤ 0.05).

**Figure 5:**
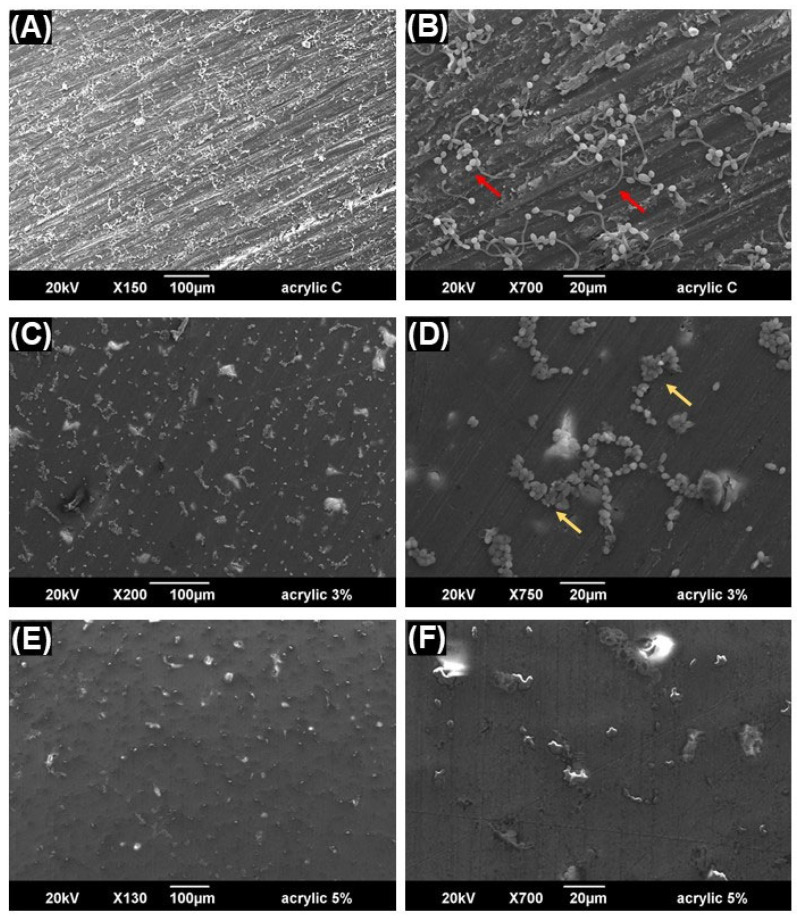
Scanning electron microscope (SEM) images of the C_12_BDMA-Cl -DBR groups (n = 1 per group). (**A**,**B**) The control DBR with no C_12_BDMA-Cl displays the growth of *Candida albicans* colonies (red arrows) over the specimens. (**C**,**D**) The 3 wt.% C_12_BDMA-Cl DBR and (**E**,**F**) the 5 wt.% C_12_BDMA-Cl DBR display reduced biofilm growth with fewer morphological yeast-to-hyphal transitions (yellow arrow) compared to the control, indicating that these colonies are less pathogenic.

In Figure 4, the *C. albicans* adhesion was significantly reduced (*p* ≤ 0.001) by approximately 1.5 to 3 log in both the 3 wt.% and 5 wt.% C_12_BDMA-Cl DBR groups, respectively. The SEM images revealed fewer *C. albicans* colonies on the 3 wt.% and 5 wt.% C_12_BDMA-Cl DBR, as illustrated in Figure 5, with fewer morphological yeast-to-hyphal transitions compared to the control. The reduction was also clearly noticeable when comparing the 3 wt.% of C_12_BDMA-Cl DBR with the 5 wt.%.

**Figure 6:**
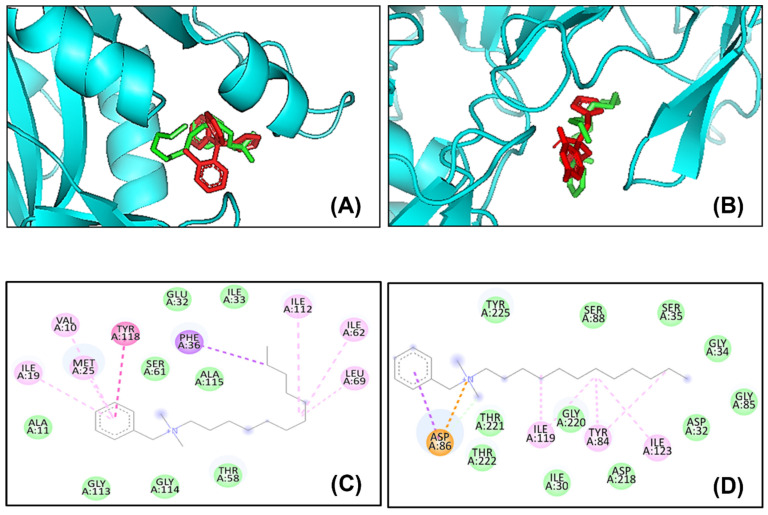
Three-dimensional (3D) structures of the binding positions between *Candida albicans* and C_12_BDMA-Cl. The C_12_BDMA-Cl compound (green) and the reference compounds (red) in the active site of (**A**) Dihydrofolate reductase (DHFR) enzyme and (**B**) Candidapepsin-1 (SAPs) enzyme. (**C**,**D**) Two-dimensional structures of the binding between C_12_BDMA-Cl compound and the two receptors; (**C**) Dihydrofolate reductase (DHFR) enzyme and (**D**) Candidapepsin-1 (SAPs) enzyme.

In Figure 6, the molecular docking results show the very good inhibition potential of C_12_BDMA-Cl as an antifungal drug. The results show that the C_12_BDMA-Cl compound can recognize the active site of the *C. albicans* pathogenic enzymes, as shown in Figure 6. The C_12_BDMA-Cl compound is green in color, and the reference compounds are red in color. Clotrimazole is the reference compound for the Dihydrofolate reductase (DHFR) enzyme (Figure 6A), and the Fluconazole compound is the reference compound for the Candidapepsin-1 (SAPs) enzyme (Figure 6B). Moreover, the molecular docking score of the binding between the C_12_BDMA-Cl compound and two *C. albicans* enzymes ranged between (−5.3) and (−5.5) kcal/mol (Table 1).

For the dihydrofolate reductase (DHFR) enzyme, the compound has a binding energy equal to −5.3 kcal/mol. This binding energy results from several interactions between the C_12_BDMA-Cl compound and the DHFR receptor, such as Pi–sigma interaction with PHE36, Pi–Pi T-shaped interaction with TYR118, Alkyl interactions with ILE112, ILE62, and LEU69, and Pi–Alkyl interactions with VAL10, ILE19, and MET25. Another expected interaction is van der Waals interactions with ALA11, GLY113, GLY114, THR58, SER61, ALA115, GLU32, and ILE33.

For the candidapepsin-1 (SAPs) enzyme, the C_12_BDMA-Cl compound has good binding energy equal to −5.5 kcal/mol with the SAPs receptor. The result shows a carbon–hydrogen bond and Pi–sigma interaction with ASP86, Alkyl interactions with ILE119 and ILE123, and Pi–Alkyl interaction with TYR84. In addition, van der Waals interactions with SER382, MET509, SER508, PHE506, GLY73, TYR72, PHE384, ALA69, MET100, ARG98, and HIS128. Figure 6C,D shows 2D structures of the binding and the type of interactions between the C_12_BDMA-Cl compound and the two receptors; Dihydrofolate reductase (DHFR) enzyme (Figure 6C) and Candidapepsin-1 (SAPs) enzyme (Figure 6D). All these interactions indicate that the C_12_BDMA-Cl compound can occupy the active sites of *C. albicans* enzymes; therefore, it has inhibition potential as an antifungal drug.

## 4. Discussion

Numerous studies have been conducted to explore different methods to overcome denture stomatitis, either by applying surface coating, immersing in cleansers, or incorporating antimicrobial fillers [5,12]. However, the integration of antimicrobial agents in polymeric materials has been preferred, as it has a slow and prolonged release in the mouth, which in turn showed therapeutic effects against *C. albicans* [32,33]. In this study, we incorporated the C_12_BDMA-Cl, which is a quaternary ammonium compound that belongs to the family of cationic surfactants. It displays strong antimicrobial activity against bacteria, fungi, and viruses [16,34]. We hypothesized that adding C_12_BDMA-Cl into DBRs would prevent the onset of denture stomatitis without compromising the mechanical properties. The hypothesis was accepted to some extent, as our results showed that the C_12_BDMA-Cl DBR, at a 3 wt.% concentration, has inhibited *C. albicans* growth without compromising the denture’s mechanical properties.

*C. albicans* is an opportunistic fungal pathogen that can adapt to different acidic conditions inside the oral cavity, including low-pH microenvironments [35,36]. A low pH can enhance the growth of *C. albicans*, especially in the presence of fermentable carbohydrates, which can lower the pH further [37]. As a result, *C. albicans* can form biofilms over different dental surfaces, causing tissue destruction and inflammation and contributing to the development of oral diseases, including denture stomatitis [38]. Additionally, in low-pH conditions, *C. albicans* can produce various factors, such as aspartyl proteinases and phospholipases, that cause tissue damage and trigger the cariogenicity of other dental pathogens, such as *Streptococcus mutans* [39,40], one of the key pathogens in dental caries development. Therefore, there is a need to design and develop different approaches to prevent the biofilm formation of *C. albicans* inside the oral cavity.

Several investigations have evaluated the antifungal properties of DBRs containing organic and inorganic compounds [9,12]. Phytochemical or phytomedical components (organic) such as Neem tree extract/powder, henna, and cinnamon (Cinnamomum zeylanicum) revealed antifungal properties when incorporated into DBRs [22,41,42,43]. Hamid et al. suggested that neem powder has displayed antifungal efficacy by reducing the adherence of *C. albicans* to DBRs [43]. In another study, Tsutsumi et al. concluded that the adhesion of *C. albicans* is diminished by the integration of the pre-reacted glass–ionomer filler in heat-polymerizing DBRs [44]. The functionalization of inorganic compounds as an antifungal approach was also attempted in several reports [12]. Particles, such as silver, titanium dioxide, silicon dioxide, zirconium dioxide, zinc oxide, and nanodiamonds were found effective in reducing the *C. albicans* growth by 50–99% when incorporated into DBRs [12].

Quaternary ammonium compounds (QACs), as polymeric materials, are well-known antiseptics because of their innate detergent, antiadhesive, and antimicrobial actions against several pathogens, including Gram-positive or Gram-negative bacteria, fungi, and specific types of viruses [45,46]. Antimicrobial QACs such as dimethylaminododecyl methacrylate (DMADDM) and dimethylaminohexadecyl methacrylate (DMAHDM), were found effective in decreasing the biofilm’s biomass and the metabolic activity of *C. albicans’* pathogenicity [10,47,48]. In this study, we incorporated C_12_BDMA-Cl into DBRs to inhibit *C. albicans* growth. In healthcare products, it has been used previously as a preservative and antiseptic in eyewashes, nasal sprays, and injectable solutions [49]. Nevertheless, its incorporation has not been studied in denture-based materials, which were the focus of this report. Here, we demonstrated that C_12_BDMA-Cl DBRs achieved a significant reduction against *C. albicans* adhesion at both concentrations, which in turn may minimize the occurrence of denture stomatitis. However, the higher concentration showed a higher fungal reduction with weakening in the mechanical and surface properties.

In this study, we conducted in silico molecular docking analysis to predict the interaction between C_12_BDMA-Cl and *C. albicans*. It was suggested that C_12_BDMA-Cl may interfere with the action of two pathogenic enzymes of *C. albicans*, DHFR and SAPs. Some studies suggest that *C. albicans* may utilize DHFR to acquire or maintain an adequate supply of folate, an essential compound for the growth and survival of *C. albicans* [50,51]. Therefore, *C. albicans* can potentially bypass the need to rely solely on the host’s folate supply. This ability to produce folate may confer an advantage to *C. albicans* in certain conditions, such as when the availability of folate is limited or when the host immune system attempts to restrict folate access to the pathogen [50,51]. On the other hand, SAPs play a crucial role in the virulence of *C. albicans* by assisting in the degradation of host proteins and facilitating tissue invasion [52]. They targets a range of host proteins, including components of the extracellular matrix, immune system molecules, and cell adhesion molecules [52]. The possible ability of C_12_BDMA-Cl to deactivate these two enzymes by occupying their active sites may contribute to the reduction in *C. albicans* in the CFU assay. Additionally, the SEM images showed that the colonies formed by *C. albicans* lacked yeast-to-hyphal transformation. This indicates that these colonies are less pathogenic and weaker compared to the ones that grew on the control samples [53].

Usually, the incorporation of bioactive agents deteriorates dental materials’ mechanical and physical properties [54,55]. Therefore, the overall mechanical assessment of newly designed bioactive materials is important to ensure at which concentration such materials can exert bioactivity without compromising their mechanical and physical performance [54,56]. In this study, the flexural strength values were reduced as the concentration of C_12_BDMA-Cl increased, as noticed between the 3 and 5 wt.% groups. However, at 3 wt.%, the flexural strength was about 70 MPa, which is above the minimally accepted flexural strength (65 MPa) values as stated by ISO: 1567 standard requirements [57]. The decrease in flexural strength values might be caused by the interference in the polymer matrix integrity that resulted from the additives in the resin base materials. When these additives aggregate, they form clusters, causing stress concentration areas within the matrix [58].

In the modulus of elasticity, we investigated the effect of C_12_BDMA-Cl DBRs to measure their ability to resist mastication stresses without causing permanent deformation [57]. Here, the 5 wt.% of C_12_BDMA-Cl DBR significantly decreased the elastic modulus, while the lower concentration (3 wt.%) showed no significant difference compared to the control group. However, at 3 and 5 wt.%, the modulus of elasticity ranges between 1800 and 2500 MPa, respectively, which complies with the minimum values recommended by the ADA specification No. 12 [57]. Our results showed no significant difference in microhardness between the different concentrations of C_12_BDMA-Cl DBRs and the control group, which means that the newly synthesized compound can resist wear and abrasion [59].

Surface roughness directly affects the oral tissue’s health, plaque accumulation, and *C. albicans* adhesion, as rougher surfaces have a larger surface area that provides more area for microbial accumulation [60]. Studies showed that the higher the surface roughness values, the higher the amount of *C. albicans* adhered to the denture base compared to polished smooth surfaces [59]. At both 3 and 5 wt.%, the surface roughness differed slightly from the control group. Nevertheless, surface roughness values increased as the C_12_BDMA-Cl concentration increased, possibly due to some loosely attached particles on the resin’s surface.

This study demonstrates the capability of C_12_BDMA-Cl at a concentration of 3 wt.% to enhance the antifungal effectiveness while maintaining the mechanical and physical properties of DBRs. Future investigations could focus on assessing additional mechanical and physical parameters, such as impact strength and color stability, and investigate antifungal properties using a multi-species biofilm model to mimic the complex oral environment. Additionally, it would be valuable to evaluate the long-term antifungal and mechanical performance of C_12_BDMA-Cl DBRs through artificial aging or cyclic fatigue models. It is also suggested that an in situ model be employed to evaluate the performance of C_12_BDMA-Cl within the oral cavity.

## 5. Conclusions

In this research, we investigated the effect of combining a quaternary ammonium-derivative molecule into denture base resin materials, which showed good potential in hindering the development of *C. albicans*. The results showed that the 3 wt.% C_12_BDMA-Cl DBR demonstrated antifungal activities without deteriorating mechanical performance, indicating its potential as a technique for preventing and treating denture stomatitis. In addition, a molecular docking study proved that the C_12_BDMA-Cl can recognize the active site of the two *C. albicans* enzymes. Moreover, the results of the molecular docking clarify the binding mechanism and the key binding residues of these interactions, which will be very useful in designing new compounds for better antifungal activities. In conclusion, C_12_BDMA-Cl is a promising antifungal agent for preventing the onset of denture stomatitis.

## Figures and Tables

**Table 1:** The molecular docking score (binding energies kcal/mol) between the C_12_BDMA-Cl compound and the *Candida albicans* enzymes.

Enzymes	PDB ID	Binding Affinity	No. of H-bonds	H-Bonding Residues	Interacted Residues	Van Der Waals Residues
Dihydrofolate reductase (DHFR)	1AI9	−5.3	-	-	PHE36, TYR118, ILE112, ILE62, LEU69, VAL10, MET25, ILE19	ALA11, GLY113, GLY114, THR58, SER61, ALA115, GLU32, ILE33
candidapepsin-1 (SAPs)	2QZW	−5.5	1	ASP86	ASP86, ILE119, TYR84, ILE123	TYR225, SER88, GLY34, GLY85, ASP32, ASP218, GLY220, ILE30, THR221, THR222

## Data Availability

The original contributions presented in the study are included in the article, further inquiries can be directed to the corresponding authors.
